# Supplementary material for: Synthesis of Novel Nicotinic Ligands with Multimodal Action: Targeting Acetylcholine α4β2, Dopamine and Serotonin Transporters
Source: Molecules. 2019 Oct 22;24(20):3808. doi: 10.3390/molecules24203808 (PMC6832503; doi:10.3390/molecules24203808)
Supplement: Supplementary file 1 [file molecules-24-03808-s001.pdf]

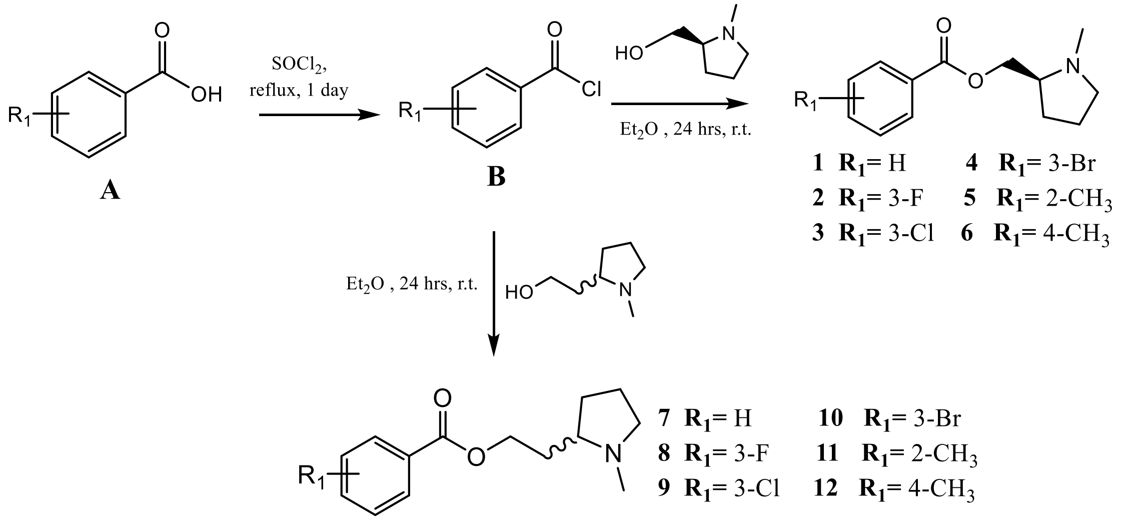

Figure S1.- Synthesis of benzoate derivatives (**1**-**12**).

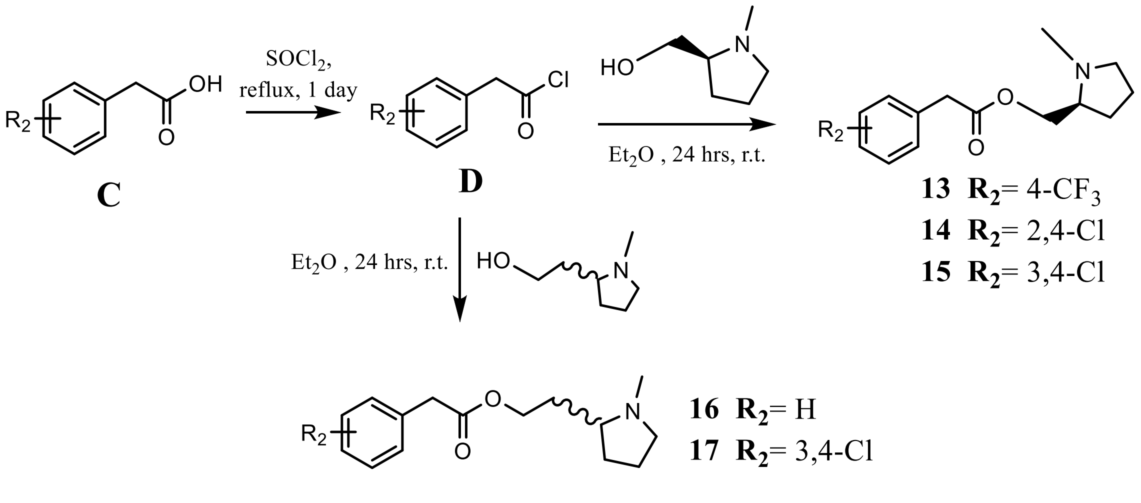

Figure S2.- Synthesis of 2-phenylacetate derivatives (**13**-**17**).

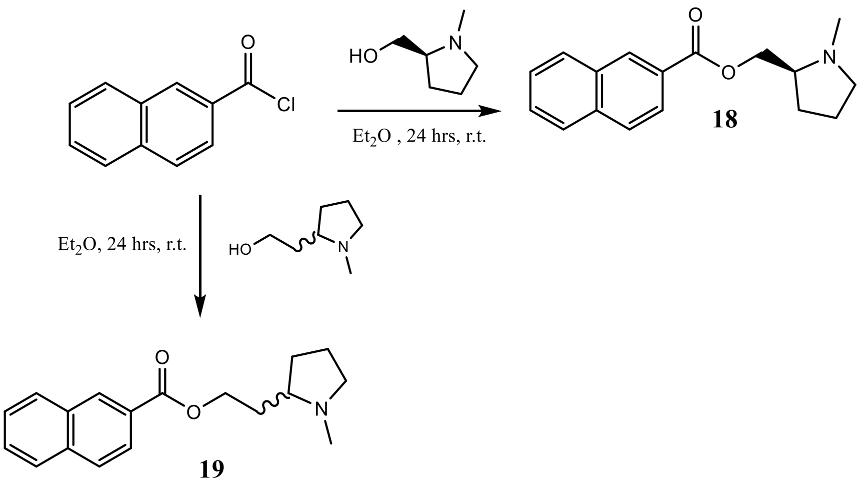

Figure S3.- Synthesis of 2-naphthoate derivatives (**18**-**19**).

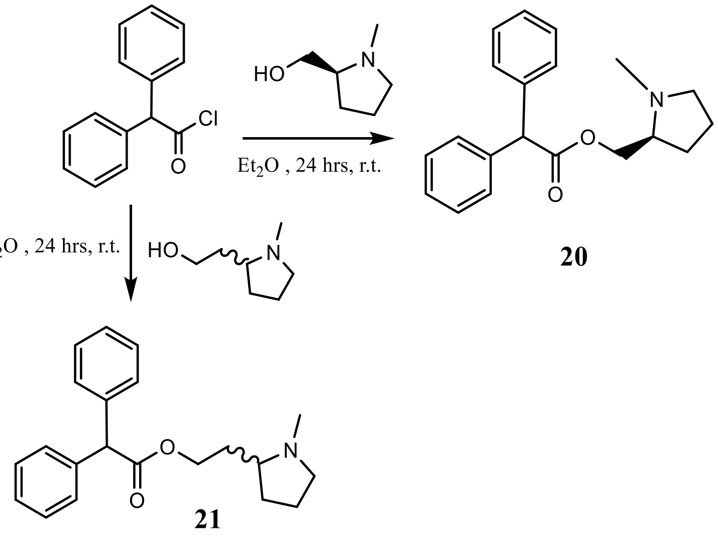

Figure S4.- Synthesis of 2,2-diphenylacetate derivatives (**20**-**21**).
